# The cooling mechanism of minuscule ribbed surfaces

**DOI:** 10.1038/s41598-020-62288-1

**Published:** 2020-03-27

**Authors:** M. Nishikawa, H. Otomo, Y. Yoshida, J. Deguchi, M. Tsukamoto, T. Yamamoto

**Affiliations:** 1 Production Technology Center, Asahi Kasei Corporation, Kawasaki, Kanagawa 210-0863 Japan; 2 R&D Laboratory for Applied Product, Asahi Kasei Corporation, Moriyama Shiga, 524-0002 Japan

**Keywords:** Mechanical engineering, Electronic devices, Computational science

## Abstract

One reason human beings wear stockings is to warm their legs. Ordinary textile materials are thermally insulative, which prevents body’s heat from dissipating. In contrary to this common sense, it was discovered that some knitted stockings made up of them permanently promote heat release and cool body. This non-intuitive phenomenon emerges when micro-size yarns are knitted to form wide spacing between neighboring yarns. However, the reason why they cool body was unclear because conventional principles of cooling garments cannot account for it. Here, in the basis of fluid-solid conjugate heat transfer analysis of natural convection, we have clarified the cooling mechanism originates from relative relationship between their geometric structure, a periodic alignment of minuscule ribs, and thermal boundary layer. Our novel finding revealed that sufficiently small ribs on the surface are exposed to steep temperature gradient within thermal boundary layer. Thereby, thermal conduction via ribs is enhanced complementarily as they are separated to guide cooler flow onto the surface. Our study provides a general insight into understanding permanent cooling mechanism on micro-size ribbed surfaces in contrast to conventional theory for heat sink, which is applicable not only to other clothes, but also to artificial devices or natural structures.

## Introduction

**Figure 1:**
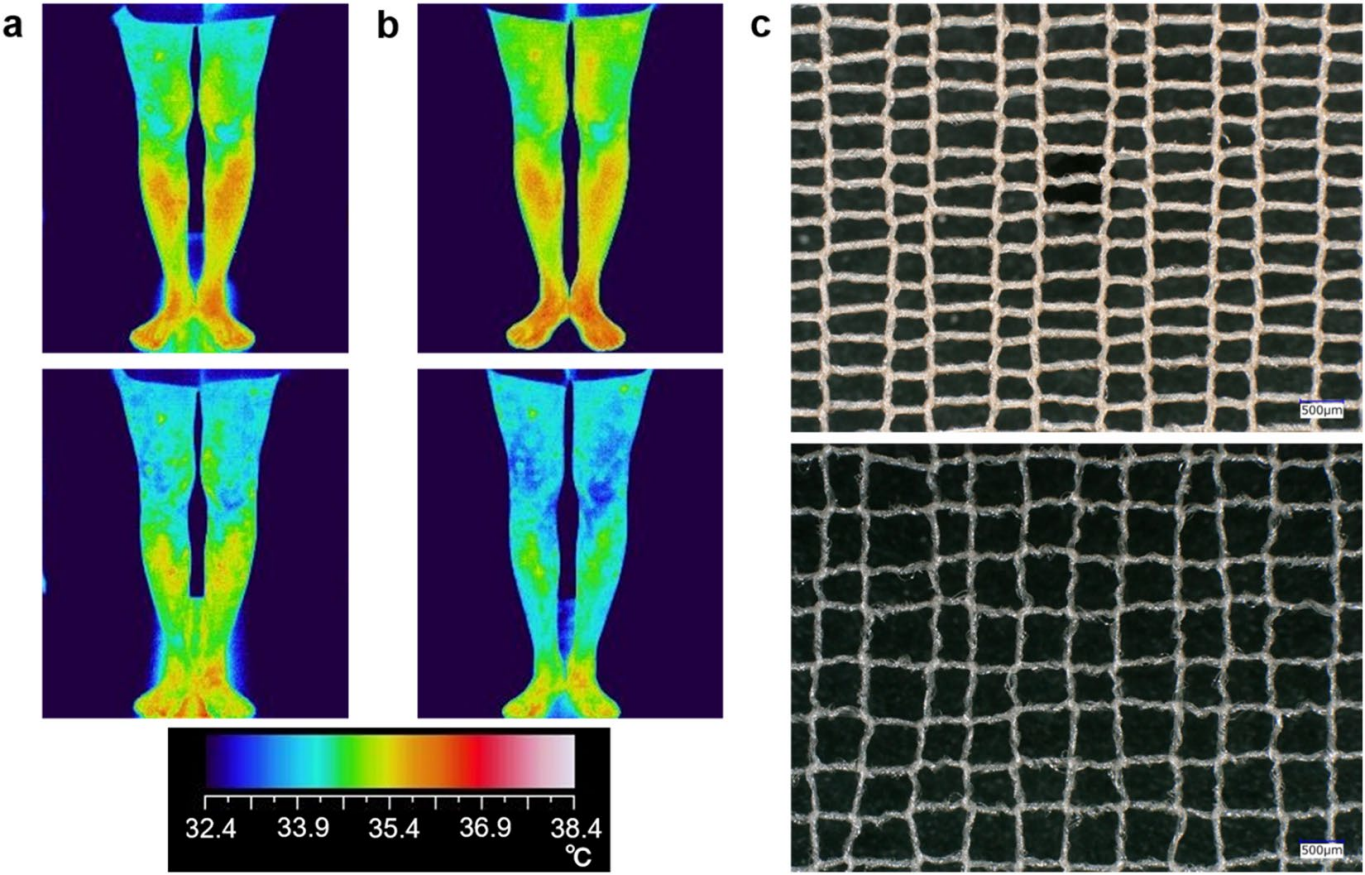
The cooling effect of knitted stockings, (**a**,**b**) change of human leg’s temperature after walking. (**a**) On bare legs, and (**b**) with cooling stockings. (**c**) Microscope images of worn stockings of commonplace (upper) and cooling (lower). Note the latter is knitted to be wider spacing between yarns along the vertical direction.

Cooling garments have been widely studied in the pursuit of various benefits for human life, particularly in terms of physical properties, such as water evaporativity, phase-transition heat, and radiation transparency^1–3^. Nowadays, despite ordinary textile materials, it was found wearing stockings, knitted as micro-size yarns (~50 μm) were separated far from each other (~400 μm), reduced human body’s temperature (see Fig. 1a,b)^4^. Even in our inorganically-designed experimental setup, where a thermal metallic body was kept stationary under chemical-equilibrium and windless conditions, 1~4% higher heat release than the unworn was steadily measured. Then, heat release efficiency is experimentally elevated as (1) spacing is widened, (2) yarn diameter is reduced. These facts indicate that endothermic reactions, thermal radiation, or motion-driven flow are unconcerned for the fundamental to cooling effect of the stockings, although they are origins of conventional passive cooling garments^1^.

The knit fabrics consist of a periodic structure of yarns^5^, and are formed to be repeated rib-like protrusions when worn tightly (see Fig. 1c). A number of previous researches revealed that ribbed surfaces promote heat transfer in forced-convection internal flow^6–9^. This is realized due to rib’s guiding huge amount of cool flow into the vicinity of the surface. On the other hand, Tanda *et al*. investigated natural convection along ribbed surfaces with optical techniques, and concluded that heat transfer augmentation by adding millimeter-size ribs they prepared was useless in a series of their studies^10–13^. Similar issue is micro-finned heat sinks. Their heat transfer coefficients were improved as fin’s height decreased and spacing increased, but some were found to be less than smooth surface’s^14,15^. In this way, it is uncertain whether ribs enhance heat transfer on natural-convection environments, which are crucial for permanent passive cooling functions. Therefore, as a model system to consider heat transfer phenomena of wearing the stockings, we investigated natural convection along vertical small ribbed surfaces based on fluid-solid conjugate heat transfer analysis.

**Figure 2:**
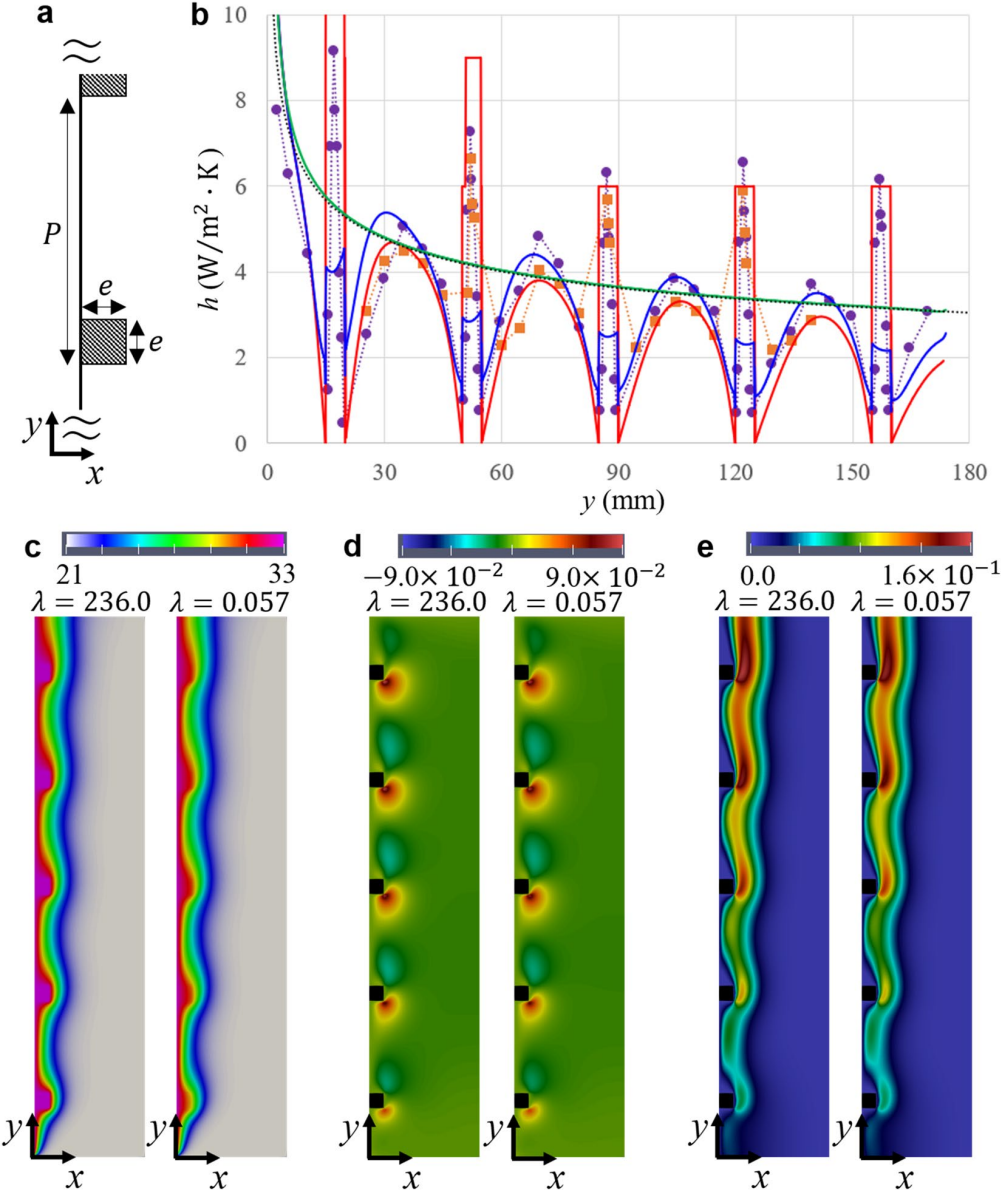
Numerical simulations of natural convection on smooth and millimeter-size ribs’, (**a**) the geometric definition of rib size *k* and pitch *P* in this study. (**b**) Local heat transfer coefficients of flat and millimeter-size ribs’. For the validation of our numerical model, experimental results, in which the aluminum ribs were used, are referred of Tanda’s (orange: holographic interferometry, purple: schlieren experiment)^11^, and corresponding numerical simulation is of aluminium thermal conductivity ${\rm{\lambda }}=236.0\,{\rm{W}}/{\rm{m}}\cdot {\rm{K}}$ (red). For comparison, textile thermal conductivity (${\rm{\lambda }}=0.057\,{\rm{W}}/{\rm{m}}\cdot {\rm{K}}$: (blue) and smooth surface’s (black: analytical solution, green: simulation) are shown. (**c–e**) Physical fields around ribbed surfaces of, (**c**) temperature, (**d**) horizontal velocity, and (**e**) vertical velocity.

Any numerical simulation requires the verification and validation to be entitled to quantitative arguments. Thus, we first simulated two examined situations, (1) smooth, and (2) a millimeter-size ribbed surfaces. Through this study, a vertical plate of height *H* = 175 mm, thickness *t* = 0.2 mm was arranged in two-dimensional external-flow configuration. With correspondence to our experiments, temperature on the wall surface *T*^*w*^ = 33 °C, and ambient temperature *T*^∞^ = 21 °C were decided. The analytical solution of natural convection on a vertical isothermal surface was formulated by Squire and Eckert^16^, for which local heat transfer coefficient *h* and thermal boundary layer thickness *δ* are respectively expressed as,

1 $$h(y)=0.508{\lambda }^{fluid}{y}^{-1}{\left(\frac{{{\rm{PrRa}}}_{y}}{0.952+{\rm{\Pr }}}\right)}^{1/4},$$

2 $$\delta (y)=3.936y{\left(\frac{0.952+{\rm{\Pr }}}{{{\rm{PrRa}}}_{y}}\right)}^{1/4},$$

where *y*, ${\lambda }^{fluid}$, Pr, and Ra_*y*_ are vertical location, thermal conductivity, Prandtl numbers, and Rayleigh numbers of fluid. According to the Eq. (2), thermal boundary layer thickness on the plate was less than 17.1 mm, for which distances between the surface and infinite boundary were prepared to be beyond it. Laminar flow was just considered since the turbulent transition criteria 10^8^ ≪ Ra_*y*_ was unsatisfied. As to ribbed conditions, five squared ribs of their edge length *e* = 5 mm were arranged with a pitch *P* = 30 mm symmetrically with respect to a center of the plate (Fig. 2a). Rib’s thermal conductivities ${\lambda }^{solid}$ were $236.0{\rm{W}}/{\rm{m}}\cdot {\rm{K}}$ and $0.057\,{\rm{W}}/{\rm{m}}\cdot {\rm{K}}$ assuming alminium and fibrous materials respectively. Note the former condition is almost the same with the experiments conducted by Tanda team. (their setup: *e* = 4.85 mm, *H* = 175 mm, *t* = 12 mm, ${T}^{w}-{T}^{\infty }=11\,^\circ {\rm{C}}$, alminium ribs)^11^. Figure 2b shows local heat transfer coefficients of our numerical simulations together with the analytical solution and the experimental data. Obviously, the theoretical prediction and our computed values for smooth surfaces accord quite well. Comparison of experiment and simulation in the ribbed case shows quantitative agreement over a broad middle area, thus we have successfully validated the quantitative capability of our method to predict heat transfer coefficients in the area. Further, temperature distributions in Fig. 2c visually resemble the images taken by a schlieren optical technique^10–12^. Compared with forced-convection internal flow on ribbed surfaces^8,9^, similar pattern of velocity distributions was observed as shown in Fig. 2d,e. In spite of external flow in our situation, outer cooler flow was guided into the vicinity of the surface. This can be explained by the fact natural-convection mainstream was just dragged by surroundings of each rib. On a different note, low-thermal-conductivity ribs promote heat transfer between neighboring ribs for the same reason with the previous studies^9,12^. Moreover, a remarkable point is that heat transfer coefficient via ribs comes to be largely below smooth surface’s, which implies millimeter-size ribs made of insulative materials work as thermal resistance.

**Figure 3:**
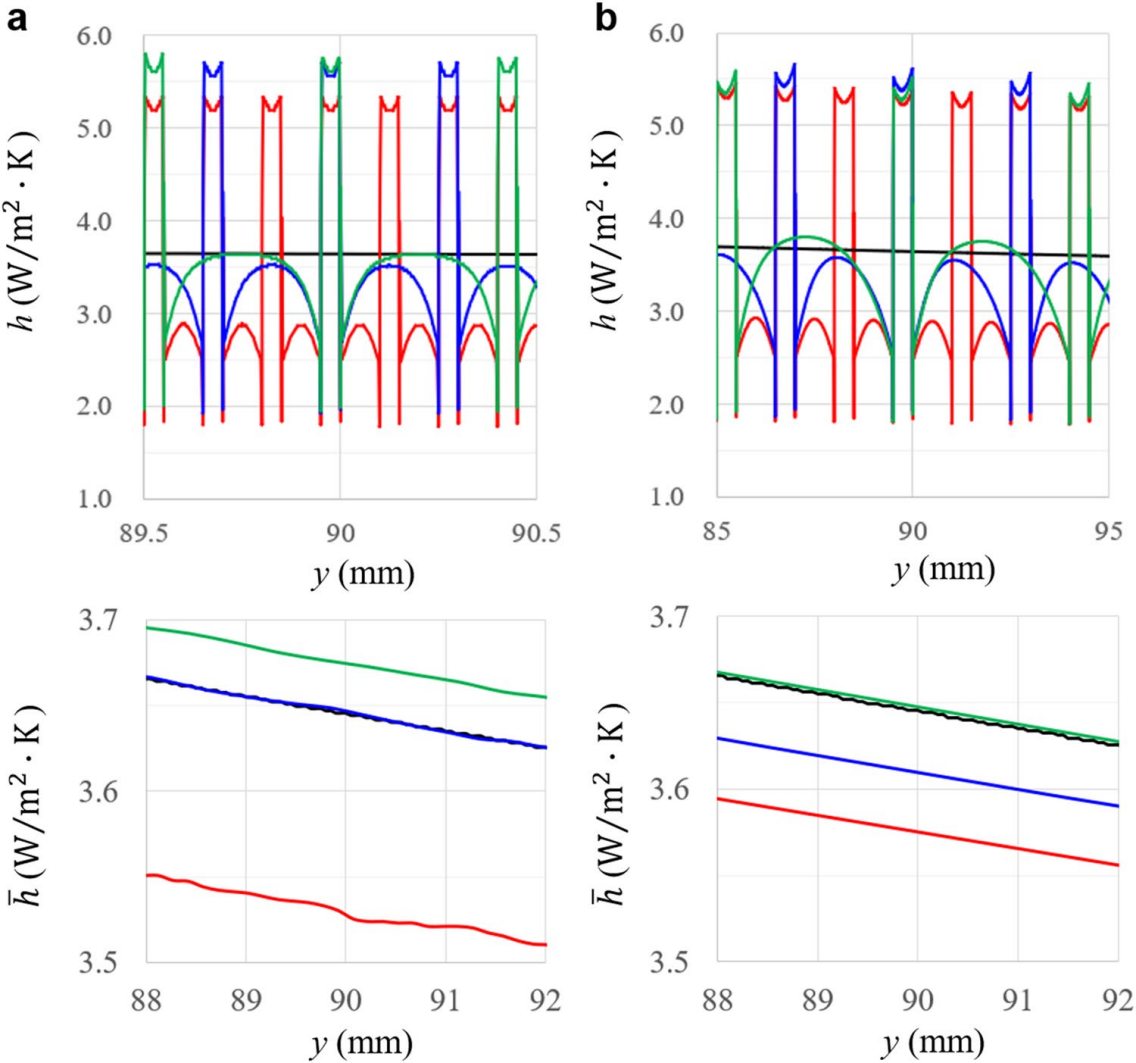
Numerical simulations of natural convection on micro-size ribs’, Local heat transfer coefficients (upper) and their averages (lower) of (**a**) *e* = 50 μm and (**b**) *e* = 500 μm. Each plot refers to smooth’s (black), *P*/*e* = 3 (red), 6 (blue), and 9 (green). The rib’s thermal conductivity is ${\rm{\lambda }}=0.057\,{\rm{W}}/{\rm{m}}\cdot {\rm{K}}$.

By means of our validated numerical method, we have investigated heat transfer on micro-size ribbed surfaces (*e* = 50, 500 μm). From the bottom edge of the plate, ribs were periodically arranged with pitch-to-size ratio *P/e* fixed (*P/e* = 3, 6, 9). Rib’s thermal conductivity was chosen of $0.057\,{\rm{W}}/{\rm{m}}\cdot {\rm{K}}$ which corresponded to our textile insulative materials. Figure 3 exemplifies local heat transfer coefficients in the middle area including several periodic unit cells, because we confirmed the same was true of the entire area. Narrow rib-rib spacing (*P/e* = 3) induced hot recirculation flow in the whole space sandwiched with neighboring ribs^17^, which led to uniformization of temperature distributions near the surfaces. Therefore, their heat transfer coefficients were entirely at a low level, particularly among the fluid area. As spacing was widened (*P/e* = 6, 9), more amount of cool flow was guided onto the surface. Accordingly, direct heat release from surface to fluid was promoted. Such a trend was also reported in the studies of millimeter-order ribs^7,12^. However, it is noteworthy that all heat releases via ribs was drastically enhanced, and exceed smooth’s despite thermally insulative properties. In addition, as rib’s size was reduced, the heat release via ribs was enhanced, whereas the release between neighboring ribs was suppressed. The latter is reasonable in that the larger ribs can take the faster and cooler natural-convection flow to the surface area. We will later discuss why the former occurs. Here, the effective heat release can be evaluated by average heat transfer coefficient $\bar{h}$. Except for the cases hot recirculation flow dominates heat transfer (*P*/*e* = 3), average heat transfer coefficients were elevated in the same trend with our sensitivity experiments of the stockings. Further, some of them exceeded the smooth surface’s, which proves the cooling effect emerged. Heat release efficiency increases by ~1%, which is valid level to account for our experimental measurements quantitatively. As a result, we have numerically elucidated that cooling mechanism of the stockings in natural convection along micro-size ribbed surfaces.

This intriguing result is greatly owing to the enhancement of heat release through minuscule ribs. In general, heat transfer phenomena are determined within thermal boundary layers. According to the Eq. (2), thermal boundary layer thickness ranges 5.1 mm to 17.1 mm in the upper 99% area on the plate, which means almost all micro-size ribs are sufficiently smaller than the thickness $(e\ll \delta )$. Therefore, addition of such small ribs is regarded as a perturbation with respect to the solution of natural-convection flow on a vertical isothermal surface, whose temperature and temperature gradient profiles are analytically described as^16^,

3 $$T(x)={T}^{\infty }+({T}^{w}-{T}^{\infty }){(1-x/\delta )}^{2},$$

4 $$\nabla T(x)=-\,2({T}^{w}-{T}^{\infty })(1-x/\delta )/\delta .$$

Quadratically temperature profile decays to ambient temperature, which means that the closer to the surface horizontal location *x* is, the steeper temperature gradient becomes. Therefore, smaller ribs are exposed to steeper temperature gradient, which results in the enhancement of heat release via ribs whose thermal conductivity is beyond fluid media, as Fourier’s law suggests a perturbative heat flux relation $q\propto -{\lambda }^{solid}\nabla T(e)$. In reality, it also has influence on convective flow around ribs as we argued, not simply proportional to each physical quantity. However, increment of pitch-to-size ratio improves the perturbation condition, and guide more cool flow onto the surface. Consequently, the cooling effect emerges due to their complementary contributions.

Our investigation indicates relative relationship between geometric structure of minuscule ribbed surface and thermal boundary layer plays essential role in the cooling mechanism, which complements conventional theory for heat sink. If size of ribs, or fins, is larger relative to thermal boundary layer of the attached wall $(\delta \ll e)$, their own boundary layer is formed on themselves. This is in the scope of the conventional theory, and the basis to promote heat release is to expand the surface area of ribs made of high thermal conductivity materials. On the other hand, relatively small ribs $(e\ll \delta )$ are subject to the boundary layer of the wall, whose heat transfer is enhanced as we discussed, that is, by reducing the rib’s size with adequate spacing. Our mechanism is generally applicable to other formations of thermal convective flows which give steep temperature gradients near the surface. Thus, it gives a guideline to design permanent cooling artifacts of other clothes as well as micro devices. Also, minuscule protrusions are often observed in self-organized structures in nature, in a similar fashion, some natural creatures might wear their own cooling garments to maintain life activity^18–20^.

## Methods

### Experiments

The yarns of knitted stocking in our experiments were made of spandex of Roica® BZ (19dtex) covered with Nylon yarns (13dtex). Its thermal conductivity was measured as $0.057\,{\rm{W}}/{\rm{m}}\cdot {\rm{K}}$ at 65%RH. Note the order of the thermal conductivity indicates that sufficiently the yarn is thermally insulative as the same with common textile materials. Because stretchable property of the spandex enables stockings to fit to adherend surfaces, ribbed surfaces are formed when they are worn.

In order to measure human body temperature change, four human subjects walked at 5 km/hour under 30 °C and 50%RH environment during 3 minutes. After bare-legs walking, the human subjects took enough rest and their body temperature was confirmed to be recovered, and then walking tests with stockings were conducted. The experimental procedures were done twice per person, then each trial was confirmed to get the same conclusion (Their representatives are shown in Fig. 1a,b). The study protocol was confirmed, and approved by the research ethics committee of Asahi Kasei Corporation because the test procedure in this study does not harm health of human subjects. All experiments were conducted in accordance with the guideline and the standard the research ethics committee established. Each subject agreed with informed consent.

Our experiments to measure heat release were conducted by thermal counter method. As a hot body imitating human legs, a sealed alminium cylindrical shell of 300 mm height, 140 mm diameter, and 5 mm thickness was prepared with a temperature controller to maintain an isothermal condition of 37 °C in the inner thermocouple, which made the surface to be 33 °C in all trials. To evaluate the worn situations, the shell was covered uniformly with the stocking. As laboratory’s environment, the room temperature was regulated to be 21 °C, the wind speed was less than 0.5 m/sec, and humidity is fixed at a constant set value. The radiation emissivity of alminium is relatively low, so that we reduce influence of radiation into heat release as possible. After reach to thermal steady state was carefully confirmed, heat releases were obtained as time-averaged measurement values of the heater’s power consumptions during a fixed period. Note that we have confirmed that the stockings made of the material we used in this study did not give rise to cooling effect on the usual knitting condition $(P/e \sim 3)$. Incidentally, our experiment showed the higher heat release as room humidity is set to be higher. The thermal conductivity of yarns increases as humidity increases, because more water is included in the yarn. Such a heat release dependence on thermal conductivity has been also reported in the research of micro heat sink^21^.

### Simulations

In our fluid-solid conjugate heat transfer analysis, the governing equations were used of the steady compressible Newtonian fluid with the equation of state as the ideal gases in fluid domains, and of the steady heat diffusion in solid domains. Physical properties of fluid were of air at 27 °C. As our simulation software, the finite volume method computational fluid dynamics solver, chtMultiRegionSimpleFoam of the openFOAM package, was used, and thus semi-implicit method for pressure linked equations (SIMPLE) method was used to solve the pressure-velocity-density coupled equations. In usual, computed spaces were equally meshed by 50 μm length squares. As to cases of micro-size ribs, meshes near the plate’s surface were gradually refined to be 6.25 μm length squares to precisely deal with micro-order phenomena. Convective terms in the governing equations were discretized by Total-variation-diminishing (TVD) cubic schemes to describe buoyant convection with the semi-third-order accuracy. Linear algebraic solvers are prepared for each field as, velocity: diagonal incomplete LU (DILU) factorization preconditioned stabilized bi-conjugate gradient (BiCG Stab) method, pressure: diagonal incomplete Cholesky (DIC) factorized generalized geometric-algebraic multigrid (GAMG) preconditioned conjugate gradient (CG) method, temperature: DIC factorized GAMG method. These linear solvers were verified to be in stable convergence in each inner iteration. Global convergences in SIMPLE iterations were attained by cautiously confirming residuals as well as computed values of local heat transfer coefficients. Local heat transfer coefficients of solid domain, heat transfer via ribs, were calculated by using temperature gradient value on the isothermal surface, which are effective estimation in thermal steady state.
